# Being Moved, Recent Stress, and Self-Reported State Resilience After Tragic Narrative Exposure: An Exploratory Secondary Analysis

**DOI:** 10.3390/bs16101936

**Published:** 2026-10-17

**Authors:** Jinwen Zhu, Qiangqiang Fan, Yang Wu

**Affiliations:** College of Arts, Northeastern University, Shenyang 110819, China; zhujinwen@mails.neu.edu.cn (J.Z.); wuyang1@mails.neu.edu.cn (Y.W.)

**Keywords:** being moved, state resilience, recent stress, tragic narrative, aesthetic emotion, mixed emotions, affective adjustment, secondary analysis

## Abstract

Tragic narratives often elicit both sadness and the feeling of being moved, but it remains unclear whether the same-session association between post-exposure being moved and post-exposure self-reported state resilience is distinguishable from co-occurring affect and differs by recent stress. This secondary analysis used the two tragic narrative conditions from a broader three-condition project. Participants (*N* = 107) viewed a tragic animated narrative or read a content-matched text narrative adapted from The Little Match Girl. State resilience was measured before and after exposure; being moved, sadness, broader affect, and recent stress were assessed in the same session. In the primary moderation model adjusting for pre-exposure resilience, the Being Moved × Recent Stress interaction reached the conventional significance threshold using ordinary least-squares standard errors (B = 0.22, *p* = 0.034; ΔR^2^ = 0.029). The estimated simple slope was positive among participants reporting recent stress (B = 0.25, *p* < 0.001) but was not distinguishable from zero among those reporting no recent stress (B = 0.04, *p* = 0.604). However, the interaction was weaker with HC3 standard errors (*p* = 0.059), the bootstrap confidence interval included zero, and the pattern was attenuated after affective adjustment and was not reproduced using the original four-level stress coding. These exploratory findings indicate a model- and coding-sensitive same-session moderation pattern rather than evidence that being moved improves resilience under recent stress.

## 1. Introduction

Tragic narratives often evoke mixed emotional responses rather than a single negative state. Stories centered on loss, suffering, attachment, and moral value can elicit sadness while also evoking being moved, a complex aesthetic emotion associated with warmth, meaning, social connectedness, and emotional stirring (Menninghaus et al., 2015; Zickfeld et al., 2019; Hanich et al., 2014; Larsen & McGraw, 2011; Berrios et al., 2015). This mixed-affective profile is central to the psychological appeal of tragic media and complicates simple interpretations of tragic narratives as either emotionally harmful or beneficial (Hanich et al., 2014; Menninghaus et al., 2019; Oliver & Raney, 2011).

Being moved has been discussed in research on aesthetic emotions and kama muta as an affective episode that is partly positive, partly negative, and often tied to perceived intensifications of communal sharing or relational significance (Menninghaus et al., 2015; Cova & Deonna, 2014; Kuehnast et al., 2014; Zickfeld et al., 2019; Herting & Schubert, 2022; Fiske et al., 2019; Seibt et al., 2017). A key question for this literature is whether being moved has psychological associations that are distinguishable from sadness and from broader positive or negative affect (Menninghaus et al., 2015, 2019; Zickfeld et al., 2019). This question is especially important in tragic narrative contexts, where being moved and sadness commonly co-occur (Hanich et al., 2014; Larsen & McGraw, 2011; Berrios et al., 2015). Recent research further indicates that being moved is a conceptually heterogeneous experience that may arise from social connection, core values, meaningful life events, and both joyful and sorrowful contexts (Hänninen & Koski-Jännes, 2025). Media-based research similarly shows that responses labelled as being moved or kama muta may involve overlapping emotional and relational components rather than a single, uniformly positive state (Möller & Strycharz, 2025). These findings reinforce the need to distinguish being moved from adjacent affective states while recognizing that complete statistical separation may not be psychologically realistic.

A companion analysis of the broader three-condition dataset found that sadness and being moved showed opposing adjusted associations with state resilience in primary models, but that the positive association involving being moved was less robust after broader positive and negative affect were considered. Those findings suggest that the association involving being moved may not be statistically separable from the broader affective configuration elicited by tragic media. This possibility is consistent with theoretical work emphasizing that aesthetic emotions are multicomponent experiences embedded within broader appraisal and affective processes (Zickfeld et al., 2019; Herting & Schubert, 2022; Menninghaus et al., 2019).

The present secondary analysis focuses on recent stress as a possible contextual boundary condition. Recent psychometric research has also emphasized the distinction between relatively stable resilience and state-sensitive resilience-related self-evaluations, while noting that many commonly used resilience measures may have limited sensitivity to short-term within-person variation (Child & Medvedev, 2024). Accordingly, the present study interprets the outcome narrowly as post-exposure self-reported state resilience rather than as observed adaptation or recovery over time. Participants who have recently experienced stress may differ in how they interpret or evaluate an emotionally meaningful narrative experience, because the associations between emotional resources and resilience-related outcomes can depend on current regulatory demands (Cohen & Wills, 1985; Razak et al., 2025; Ringgold & Rohleder, 2025; Egan et al., 2024). However, recent stress was not experimentally manipulated and was assessed with a single self-report item. Accordingly, the present study treats recent stress as a contextual moderator of same-session self-reported associations, not as evidence of a causal stress-buffering mechanism.

Existing research has examined being moved in meaningful media experiences and has separately investigated the relationships among affect, stress, and resilience. However, limited evidence directly addresses whether the association between post-exposure being moved and post-exposure self-reported state resilience varies as a function of recent stress. The novelty of the present secondary analysis lies in examining recent stress as a potential contextual boundary condition and in evaluating whether the estimated interaction remains consistent after adjustment for co-occurring affect and under alternative stress coding. The primary research question was whether recent stress moderated the association between post-exposure being moved and post-exposure self-reported state resilience after adjustment for pre-exposure resilience. Additional research questions examined whether this interaction remained after adjustment for co-occurring post-exposure sadness and broader positive and negative affect, and whether it was reproduced using the original four-level recent-stress variable. Given the observational measurement of recent stress, the same-session assessment, and the non-preregistered secondary-analysis design, these analyses were treated as exploratory and hypothesis-generating rather than as a confirmatory test of a causal stress-buffering mechanism.

## 2. Materials and Methods

### 2.1. Participants

The present study reports analyses from a broader research project that initially recruited 200 adults aged 18 to 45 years. The broader project included three conditions: an animated narrative condition, a text-based narrative condition, and a neutral documentary control condition. Thirty-six participants were excluded because they did not meet the prespecified eligibility or data-quality criteria. Eligibility criteria required participants to be 18–45 years old, have normal or corrected-to-normal vision and hearing and adequate reading comprehension, report no history of psychiatric or neurological disorders or current use of psychotropic medication, and pass the visual-imagery screening. Data-quality criteria required participants to pass the embedded attention and material-comprehension checks and to comply with the stimulus-exposure instructions. This resulted in 164 valid participants in the broader dataset. Because the present analysis focused on the two tragic narrative conditions, the 57 participants in the neutral documentary control condition were excluded, yielding a final analytic sample of 107 participants (56 female, 51 male; mean age = 28.4 years, SD = 7.1, range = 19–45), with 53 in the animation group and 54 in the text group.

All participants were fluent in the language of the study materials and provided informed consent prior to participation. Ethical approval for the broader project was granted by the Biomedical and Medical Ethics Committee of Northeastern University, Shenyang, China (Approval No. NEU-EC-2026B073S).

### 2.2. Data Source and Analytic Scope

The data were drawn from a broader three-condition project. The present manuscript and a companion manuscript share the same broader dataset, the same tragic-narrative subsample, and several variables. However, the two manuscripts address distinct research questions. The companion manuscript used the full three-condition sample to examine the adjusted associations of sadness and being moved with state resilience, whereas the present secondary analysis uses only the two tragic-narrative conditions to examine recent stress as a potential moderator of the association between post-exposure being moved and post-exposure self-reported state resilience. The overlap in the dataset and variables is disclosed for transparency. The present secondary analysis was not preregistered and should be regarded as exploratory.

### 2.3. Procedure

Participants completed the study online during the year-end period around Chinese New Year (between 1 February 2026 and 14 February 2026) on Credamo. The survey included background and eligibility items, including visual-imagery screening, recent stress, psychiatric or neurological history and psychotropic medication use, demographic information, baseline emotion ratings, and pre-exposure state resilience. Participants were then randomly assigned to one of the three conditions in the broader project: an animated tragic narrative, a text-based tragic narrative, or a neutral documentary control condition. The present analysis used only the two tragic narrative conditions.

After stimulus exposure, participants completed several measures included in the broader project. Of direct relevance to the present analysis, post-exposure state resilience was assessed before the post-exposure emotion items. Participants subsequently completed the embedded attention, material-comprehension, and stimulus-compliance checks.

### 2.4. Materials

The tragic animated stimulus was Disney’s The Little Matchgirl (Allers, 2006), a short animated adaptation of Andersen’s story lasting approximately 6–7 min. The text stimulus was a Chinese text-based narrative adapted from Ye Junjian’s translation of Andersen’s The Little Match Girl and contained approximately 3272 Chinese characters. The animation and text shared the same core plot and event sequence, including the child’s suffering, visions of warmth and attachment, and reunion with her grandmother. The materials were presented through the online survey platform, and participants were randomly assigned to condition by the platform. No separate pilot study was conducted before the main experiment to validate the emotional response profiles of the materials.

### 2.5. Measures

#### 2.5.1. State Resilience

State resilience was measured using the State Resilience Scale (SRS) (Hiew, 2002, 2006). The SRS is a 15-item self-report measure intended to assess state-like resilience resources at the present time. Items are rated on a 5-point Likert scale from 1 (completely disagree) to 5 (completely agree). In the present study, the scale was used to operationalize participants’ momentary adaptive coping resources before and after narrative exposure. Internal consistency in the present analytic sample was excellent (pre-exposure α = 0.900; post-exposure α = 0.947).

Because the present study focused on within-session change in momentary resilience rather than stable trait resilience, the SRS was selected as a state-sensitive measure. Its strong internal consistency in the present sample supports its use for the current pre-post design, although future research should further validate the observed pattern using additional resilience measures.

#### 2.5.2. Being Moved

Being moved was assessed with a single item administered in Chinese and translated as “To what extent do you feel moved at this moment?”, rated on a 5-point scale from 1 (very slightly or not at all) to 5 (extremely). This item was administered both before and after narrative exposure to capture baseline and post-exposure levels. In the present analyses, post-exposure being moved served as the focal predictor. The original Chinese expression may encompass being moved, feeling touched, and being emotionally stirred. All measures were administered in Chinese, and no formal back-translation procedure was conducted. The single-item approach minimized participant burden in a multi-scale online design, but a multi-item measure such as the KAMMUS would provide greater reliability and construct specificity.

#### 2.5.3. Sadness

Sadness was assessed before and after narrative exposure with a single item administered in Chinese and translated as “To what extent do you feel sad at this moment?”, rated on a 5-point scale from 1 (very slightly or not at all) to 5 (extremely). Post-exposure sadness was included as a covariate in the affective-specificity analysis, whereas pre-exposure sadness was included in the baseline-emotion-adjusted model.

#### 2.5.4. Recent Stress

Recent stress was assessed with a single item asking whether participants had experienced a notable stressful event in the past month. The original response categories were no stress, mild stress, moderate stress, and severe stress. In the present analytic sample, 49 participants reported no recent stress, 43 reported mild stress, 13 reported moderate stress, and 2 reported severe stress. Because the moderate- and severe-stress categories were sparse, particularly the severe-stress category, a post hoc pragmatic recoding was adopted for the present secondary analysis. The primary models contrasted no recent stress (0; *n* = 49) with any recent stress (1; *n* = 58). Although this recoding avoided estimating separate effects from extremely small upper-category groups, it reduced information about stress severity and changed the substantive interpretation of the moderator from level of recent stress to the reported presence versus absence of a notable recent stressor. It may also have altered statistical power and the estimated form of the interaction. The originally collected four-level variable was therefore retained as an ordinal predictor in a sensitivity analysis and was given substantial weight in the interpretation. Because the interaction was not statistically significant under the original four-level coding, findings based on the binary coding should be regarded as exploratory and coding-sensitive.

#### 2.5.5. Broader Positive and Negative Affect

Broader affective states were assessed in Chinese using the International Positive and Negative Affect Schedule Short Form (I-PANAS-SF) (Thompson, 2007). Positive affect was computed as the mean of five post-exposure items: determined, alert, inspired, attentive, and active. Negative affect was computed as the mean of five post-exposure items: upset, ashamed, nervous, afraid, and hostile. The focal sadness item was analyzed separately and was not part of the negative-affect composite. In the present analytic sample, internal consistency was acceptable for positive affect (α = 0.727) and negative affect (α = 0.791).

### 2.6. Statistical Analysis

The primary analysis was a moderated multiple regression in which post-exposure self-reported state resilience served as the outcome variable. Pre-exposure state resilience was entered as a covariate to adjust for baseline differences. Post-exposure being moved was mean-centered and entered with recent stress (0 = no recent stress, 1 = any recent stress) and their interaction term. Significant interactions were decomposed using simple slope analysis (Hayes, 2022). To evaluate robustness, additional models adjusted for narrative condition, pre-exposure being moved and pre-exposure sadness, post-exposure sadness, broader post-exposure positive affect and the negative-affect composite (which did not include the separate focal sadness item), and the combination of sadness and broader affect. A sensitivity analysis retained the original four-level recent stress variable as an ordinal predictor. Additional checks used HC3 heteroscedasticity-consistent standard errors, Cook’s distance to identify influential cases, and a nonparametric bootstrap 95% confidence interval based on 5000 resamples for the primary interaction. Cases with Cook’s distance greater than 4/*N* were flagged for sensitivity analysis only; eight cases were identified, yielding *n* = 99 after exclusion. Regression assumptions were evaluated using residual-versus-fitted and Q–Q plots, variance inflation factors, the Breusch–Pagan test of heteroscedasticity, and the Ramsey RESET test of functional form. Given the modest sample size and the observed departure from normality in the lower tail of the residual distribution, HC3 heteroscedasticity-consistent standard errors and nonparametric bootstrap confidence intervals were retained as robustness checks. To contextualize the descriptive difference between pre- and post-exposure state-resilience scores, an additional paired-samples *t*-test was conducted, with the mean difference, 95% confidence interval, and Cohen’s dz reported. The proportions of participants scoring at the scale maximum before and after exposure were also examined to assess possible ceiling effects.

## 3. Results

### 3.1. Descriptive Statistics and Preliminary Analyses

Descriptive statistics and bivariate correlations are presented in Table 1. Pre-exposure and post-exposure resilience were moderately correlated (r = 0.52, *p* < 0.001). Being moved showed a positive correlation with post-exposure resilience (r = 0.33, *p* < 0.001) and small, non-significant correlations with pre-exposure resilience (r = 0.19, *p* = 0.056) and recent stress (r = 0.15, *p* = 0.123). Recent stress was not significantly correlated with either resilience measure at the bivariate level.

Mean post-exposure state-resilience scores were descriptively lower than pre-exposure scores (M = 4.08 vs. 4.18). However, the mean within-session change was small and was not statistically distinguishable from zero, M_diff = −0.10, 95% CI [−0.21, 0.01], t(106) = −1.72, *p* = 0.088, Cohen’s d_z = −0.17. This aggregate comparison addresses a different question from the moderation analysis, which examined individual differences in post-exposure self-reported state resilience after adjustment for pre-exposure resilience. Scores at the scale maximum were rare both before (0%) and after exposure (0.9%), providing little evidence of a pronounced ceiling effect.

### 3.2. Primary Moderation Analysis

In the primary moderation model, the Being Moved × Recent Stress interaction reached the conventional significance threshold using ordinary least-squares standard errors (B = 0.22, SE = 0.10, t = 2.15, *p* = 0.034, 95% CI [0.02, 0.41]). In this specification, the estimated association between being moved and post-exposure self-reported state resilience differed by binary recent-stress status. The interaction accounted for an additional 2.9% of variance beyond the main effects (ΔR^2^ = 0.029; Cohen’s f^2^ = 0.045), indicating a small effect. Because subsequent robustness analyses yielded less consistent evidence, this result is interpreted as a preliminary moderation pattern rather than as evidence of a definitive stress-contingent process. Full coefficients are reported in Table 2.

### 3.3. Simple-Slope Analysis

**Figure 1:**
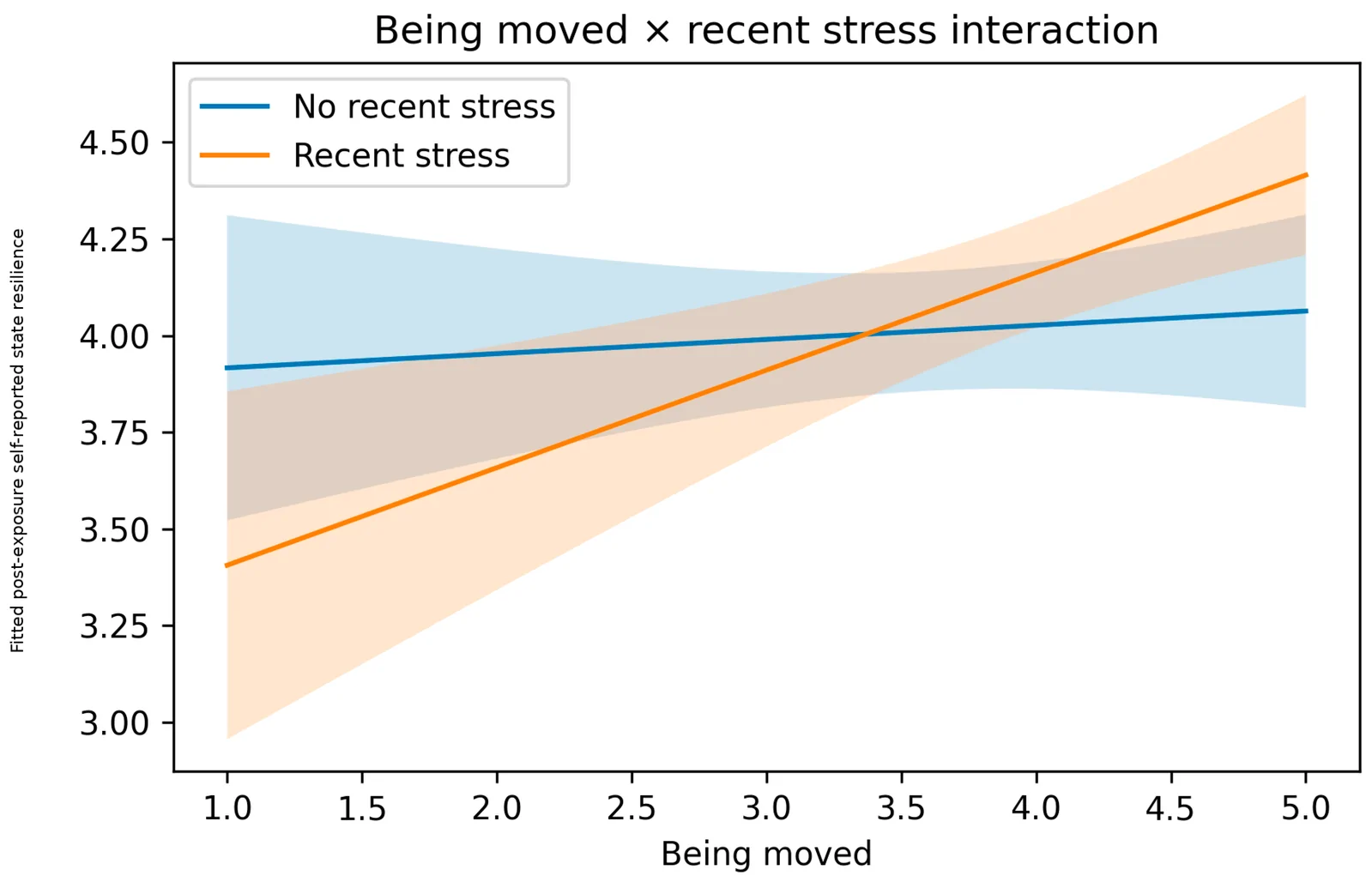
Fitted post-exposure self-reported state resilience as a function of being moved and recent stress, controlling for pre-exposure resilience (held at its sample mean). Solid lines represent fitted regression slopes for participants reporting recent stress (*n* = 58) and no recent stress (*n* = 49). Shaded bands indicate 95% confidence intervals around the fitted mean estimates.

Simple-slope estimates were examined to describe the form of the interaction in the primary model (Table 3 and Figure 1). Among participants reporting recent stress, the estimated slope relating being moved to post-exposure self-reported state resilience was positive (B = 0.25, SE = 0.07, t = 3.46, *p* < 0.001, 95% CI [0.11, 0.40]). Among participants reporting no recent stress, the corresponding slope was not statistically distinguishable from zero (B = 0.04, SE = 0.07, t = 0.52, *p* = 0.604, 95% CI [−0.10, 0.18]). These conditional estimates describe the interaction observed in the primary specification; they do not demonstrate that being moved increased resilience among participants reporting recent stress. The pattern should therefore be interpreted together with the robustness and sensitivity analyses reported below.

### 3.4. Robustness and Sensitivity Analyses

Regression diagnostics indicated no serious multicollinearity (VIFs = 1.07–2.04), no evidence of heteroscedasticity (Breusch–Pagan LM = 3.17, *p* = 0.529), and no indication of substantial functional-form misspecification (Ramsey RESET F(2, 100) = 1.30, *p* = 0.277). The residual-versus-fitted plot did not show a pronounced funnel pattern. However, inspection of the Q–Q plot indicated a departure from normality concentrated in the lower tail of the residual distribution. The result based on conventional standard errors was therefore interpreted alongside the HC3 and bootstrap analyses reported below.

A condition-adjusted model (Table 4) first examined whether the primary interaction was explained by narrative format. Narrative condition did not significantly predict post-exposure resilience (B = 0.02, *p* = 0.879), and the Being Moved × Recent Stress interaction remained significant with similar magnitude (B = 0.22, *p* = 0.035). A baseline-emotion-adjusted model additionally controlled pre-exposure being moved and pre-exposure sadness; the interaction remained similar in this model (B = 0.216, SE = 0.100, *p* = 0.032).

To evaluate whether the primary interaction reflected a being-moved-specific pattern or broader same-session affective overlap, additional models adjusted for co-occurring emotional responses (Table 5). When post-exposure sadness was added to the model, the Being Moved × Recent Stress interaction was attenuated and no longer significant (B = 0.115, SE = 0.091, t = 1.26, *p* = 0.210). Post-exposure sadness was negatively associated with post-exposure resilience (B = −0.213, *p* < 0.001).

A second robustness model adjusted for broader post-exposure positive affect and the post-exposure negative-affect composite, which did not include the separate focal sadness item. In this model, the interaction was again nonsignificant (B = 0.062, SE = 0.062, t = 1.01, *p* = 0.317). Post-exposure positive affect was positively associated with post-exposure resilience (B = 0.450, *p* < 0.001), whereas the post-exposure negative-affect composite was negatively associated with post-exposure resilience (B = −0.470, *p* < 0.001). When post-exposure sadness, positive affect, and the negative-affect composite were entered together, the interaction remained nonsignificant (B = 0.060, SE = 0.062, t = 0.96, *p* = 0.338).

Additional diagnostics indicated that the primary interaction was sensitive to the inferential method. With HC3 heteroscedasticity-consistent standard errors, the interaction was weaker and did not reach the conventional 0.05 threshold (SE = 0.113, *p* = 0.059). The nonparametric bootstrap 95% confidence interval for the interaction also included zero [−0.010, 0.427]. Cook’s distance identified eight cases above the 4/*N* threshold; when these cases were excluded as a sensitivity analysis (*n* = 99), the interaction remained positive and statistically significant (B = 0.129, SE = 0.053, *p* = 0.016), although the coefficient was smaller.

Finally, when the originally collected four-level recent-stress variable was retained as an ordinal predictor, the Being Moved × Recent Stress interaction was not statistically significant (B = 0.105, SE = 0.067, t = 1.57, *p* = 0.119, 95% CI [−0.03, 0.24]). This sensitivity analysis indicates that the observed moderation pattern depended partly on the binary operationalization of recent stress.

Taken together, the interaction reached the conventional significance threshold in the primary binary-stress model but was not consistently reproduced across affect-adjusted models, robust inference, bootstrap uncertainty estimation, and the original four-level stress coding. The interaction should therefore be regarded as preliminary, model-dependent, and sensitive to the operationalization of recent stress.

## 4. Discussion

The present secondary analysis provides preliminary evidence that recent stress may qualify the association between post-exposure being moved and post-exposure self-reported state resilience. In the primary model, this association was stronger among participants reporting recent stress than among those reporting no recent stress. The pattern remained similar after adjustment for narrative condition and baseline emotional states, suggesting that it was not attributable solely to presentation format or pre-existing differences in being moved and sadness. At the same time, the interaction was sensitive to affective adjustment, inferential method, and the operationalization of recent stress. These findings identify a potentially meaningful stress-contingent pattern while also defining the conditions under which that pattern requires further confirmation.

### 4.1. Affective Specificity and Overlap

The attenuation of the interaction after affective adjustment is theoretically informative but does not permit a simple conclusion about the psychological specificity of being moved. Being moved is a mixed and multifaceted emotional experience that may include warmth, sadness, social connectedness, meaning, moral evaluation, and bodily arousal (Menninghaus et al., 2015; Zickfeld et al., 2019; Herting & Schubert, 2022; Fiske et al., 2019; Seibt et al., 2017). Consequently, adjusting for sadness and broader affect may remove variance that is theoretically constitutive of being moved as well as variance attributable to shared affective processes.

The adjusted models therefore provide evidence of limited statistical specificity, but they do not establish that being moved lacks a distinct psychological function. Given the use of a single Chinese-language item and same-session self-report measures, the present design cannot distinguish among conceptual overlap, measurement imprecision, shared method variance, statistical overadjustment, and genuinely shared affective mechanisms. At most, the results suggest that the observed moderation pattern was embedded in a broader affective configuration rather than clearly separable from it.

### 4.2. Narrative Context and Media Format

The narrative-condition robustness model indicated that the primary interaction was not explained by the distinction between animation and text conditions. This should not be interpreted as evidence that the association generalizes across all media formats. Rather, the current data suggest that, within two versions of the same tragic narrative, the observed association was not attributable to presentation format alone. In this sense, animation and text functioned as two media instantiations of a shared tragic narrative context that elicited mixed emotional responses. This interpretation is consistent with evidence that being moved can be elicited through both audiovisual and text-based narrative materials (Hanich et al., 2014; Möller & Strycharz, 2025).

### 4.3. Implications for Future Research

The present findings point to the importance of studying aesthetic emotions within their broader emotional and regulatory context. Future studies should use validated multi-item measures of being moved (Zickfeld et al., 2019), independent measures of meaning and social connectedness, validated stress inventories (Cohen et al., 1983; Ringgold & Rohleder, 2025), delayed resilience outcomes, and designs that experimentally manipulate either stress context or narrative-evoked emotional intensity. Such work would be needed before stronger claims can be made about whether being moved has a distinct adaptive function in tragic media experiences.

### 4.4. Limitations and Future Directions

Several limitations constrain the interpretation of these findings. First, post-exposure being moved, sadness, broader affect, and post-exposure state resilience were all assessed through a same-session self-report, and recent stress was also measured within the same study session. The observed moderation pattern may therefore reflect momentary self-evaluative associations, common-method variance, response style, temporal overlap, or semantic overlap rather than actual short-term adaptation or causal changes in resilience. The use of HC3 standard errors and bootstrap confidence intervals further indicates that the primary interaction should be interpreted cautiously. In addition, post-exposure resilience was assessed before the focal post-exposure emotion ratings. Therefore, the measurement sequence does not support a temporal interpretation in which being moved preceded or produced changes in resilience.

Second, recent stress was assessed with a single item, and the original four-level variable was dichotomized for the primary analysis because only 13 participants reported moderate stress and 2 reported severe stress. This was a post hoc pragmatic recoding adopted for the present secondary analysis rather than an originally specified operationalization. Although dichotomization avoided estimates based on extremely sparse higher-stress categories, it also reduced information about stress severity and changed the substantive meaning of the moderator from the degree of recent stress to the presence versus absence of any reported stress. Importantly, the interaction was not significant when the original ordinal coding was retained. The primary moderation pattern should therefore be regarded as sensitive to how recent stress was operationalized, and the four-level sensitivity analysis places an important constraint on its interpretation.

Third, being moved was measured with a single item administered in Chinese. The original Chinese expression may encompass being moved, feeling touched, and being emotionally stirred. Future work should use validated multi-item measures such as the KAMMUS and report formal translation or back-translation procedures when cross-language emotion constructs are involved.

Fourth, the attenuation of the interaction after controlling sadness and broader affect indicates that the present data cannot establish a being-moved-specific association independent of the broader affective context. At the same time, because broader affect overlaps conceptually with components of being moved, these adjusted models should be interpreted as tests of statistical specificity rather than definitive separations among emotion constructs.

Fifth, the present analysis did not include the neutral documentary control condition from the broader project. The analytic focus on the two tragic narrative conditions was appropriate for the research question, but it limits the ability to attribute observed associations specifically to tragic narrative exposure rather than to same-session measurement or other time-varying factors. The companion manuscript addresses group-level comparisons involving the control condition.

Sixth, the relatively high pre-exposure state-resilience scores also raise the possibility of some range restriction. Although post-exposure scores were descriptively lower, the mean within-session change was small and not statistically distinguishable from zero. Exact maximum scores were rare at both assessments, providing little evidence of a pronounced ceiling effect in the strict sense. Nevertheless, the concentration of scores toward the upper end of the scale may have reduced variability and limited sensitivity to small within-session differences.

Seventh, no a priori power analysis was conducted specifically for the present secondary moderation analysis. The modest analytic sample, sparse moderate- and severe-stress categories, and small estimated interaction effect limited statistical precision. This limitation is illustrated by the contrast between the result based on conventional standard errors and those based on HC3 standard errors and bootstrap uncertainty. Interpretation should therefore place greater weight on the effect estimate, confidence intervals, and consistency across model specifications than on whether an individual *p* value falls immediately above or below 0.05.

## 5. Conclusions

This secondary analysis provides preliminary evidence that recent stress may qualify the association between post-exposure being moved and post-exposure self-reported state resilience. In the primary binary-stress model, the estimated association was positive among participants reporting recent stress but was not statistically distinguishable from zero among those reporting no recent stress. However, the interaction was sensitive to adjustment for co-occurring affect, robust-standard-error and bootstrap-based inference, and the operationalization of recent stress. The findings therefore identify a potentially meaningful stress-contingent pattern rather than demonstrating that being moved increases resilience or operates as an independent stress-buffering mechanism. Preregistered studies with more precise measures, temporally separated assessments, and adequately powered samples are needed to determine whether this pattern can be replicated and clarified.

## Figures and Tables

**Table 1:** Descriptive statistics and bivariate correlations (*N* = 107).

Variable	M	SD	1	2	3	4
1. Pre-resilience	4.18	0.50	—			
2. Post-resilience	4.08	0.66	0.52 ***	—		
3. Being moved	3.78	1.06	0.19	0.33 ***	—	
4. Recent stress	0.54	0.50	−0.17	0.02	0.15	—

Note. *** *p* < 0.001. Recent stress coded as 0 = no, 1 = yes.

**Table 2:** Primary moderation model for post-exposure self-reported state resilience (*N* = 107).

Predictor	B	SE	t	*p*	95% CI
Intercept	1.39	0.48	2.90	0.005	[0.44, 2.35]
Pre-resilience	0.63	0.11	5.71	<0.001	[0.41, 0.85]
Being moved	0.04	0.07	0.52	0.603	[−0.10, 0.18]
Recent stress	0.09	0.11	0.83	0.410	[−0.13, 0.31]
Moved × Stress	0.22	0.10	2.15	0.034	[0.02, 0.41]

Note. R^2^ = 0.356, adjusted R^2^ = 0.331, F(4, 102) = 14.12, *p* < 0.001. The interaction term added ΔR^2^ = 0.029 beyond the main-effects model, corresponding to Cohen’s f^2^ = 0.045. Recent stress coded as 0 = no, 1 = yes. Being moved was mean-centered prior to analysis.

**Table 3:** Conditional effects of being moved on post-exposure resilience at values of recent stress.

Stress	B	SE	t	*p*	95% CI
No stress (0)	0.04	0.07	0.52	0.604	[−0.10, 0.18]
Stress (1)	0.25	0.07	3.46	<0.001	[0.11, 0.40]

Note. Conditional effects derived from the moderation model in Table 2.

**Table 4:** Condition-adjusted moderation model for post-exposure self-reported state resilience (*N* = 107).

Predictor	B	SE	t	*p*	95% CI
Intercept	1.39	0.48	2.89	0.005	[0.44, 2.35]
Pre-resilience	0.63	0.11	5.65	<0.001	[0.41, 0.85]
Being moved	0.03	0.07	0.48	0.630	[−0.11, 0.18]
Recent stress	0.08	0.11	0.77	0.444	[−0.13, 0.30]
Moved × Stress	0.22	0.10	2.14	0.035	[0.02, 0.42]
Condition (animation)	0.02	0.11	0.15	0.879	[−0.20, 0.24]

Note. R^2^ = 0.357, adjusted R^2^ = 0.325, F(5, 101) = 11.20, *p* < 0.001. Condition coded as 0 = text, 1 = animation. Recent stress coded as 0 = no, 1 = yes. Being moved was mean-centered prior to analysis.

**Table 5:** Robustness and sensitivity models for post-exposure self-reported state resilience.

Model	Adjustment/Coding	B	SE	t	*p*	95% CI	R^2^
Primary	Pre-resilience	0.216	0.100	2.15	0.034	[0.02, 0.41]	0.356
Condition-adjusted	Narrative condition	0.217	0.101	2.14	0.035	[0.02, 0.42]	0.357
Baseline-emotion-adjusted	Pre-being moved + pre-sadness	0.216	0.100	2.17	0.032	[0.02, 0.41]	0.390
Sadness-adjusted	Post-exposure sadness	0.115	0.091	1.26	0.210	[−0.07, 0.29]	0.501
Affect-adjusted	Positive affect + negative-affect composite	0.062	0.062	1.01	0.317	[−0.06, 0.18]	0.771
Fully adjusted	Sadness + broader affect	0.060	0.062	0.96	0.338	[−0.06, 0.18]	0.771
HC3 primary model	HC3 robust SE	0.216	0.113	1.91	0.059	—	0.356
Influential cases removed	Cook’s D ≤ 4/*N*	0.129	0.053	2.45	0.016	[0.02, 0.23]	0.616
Ordinal stress	Original four-level stress	0.105	0.067	1.57	0.119	[−0.03, 0.24]	0.344

Note. B (SE) reports the interaction coefficient and its standard error. The primary, condition-adjusted, baseline-emotion-adjusted, sadness-adjusted, affect-adjusted, fully adjusted, HC3, and influential-case models used binary recent stress coding (0 = no recent stress, 1 = any recent stress). The ordinal-stress model retained the original four-level stress variable. The focal sadness item was analyzed separately and was not part of the negative-affect composite. All models used *N* = 107 except the influential-case sensitivity model (*n* = 99). The bootstrap 95% confidence interval for the primary interaction was [−0.010, 0.427]. R^2^ values are rounded to three decimals.

## Data Availability

The data presented in this study are available on request from the corresponding author due to institutional and ethical restrictions concerning participant data.
